# Subacute Stage of *Encephalitozoon cuniculi* Infection in Eye Lesions of Rabbit in Turkey

**Published:** 2018

**Authors:** Özcan ÖZKAN, Mehmet Eray ALCIGIR

**Affiliations:** 1. Dept. of Biology, Faculty of Science, Çankırı Karatekin University, Çankırı, Turkey; 2. Dept. of Pathology, Faculty of Veterinary Medicine, Kırıkkale University, Kırıkkale, Turkey

**Keywords:** *Encephalitozoon cuniculi*, Eye lesions, Clinicopathology, Subacute findings

## Abstract

**Background:**

*Encephalitozoon cuniculi* is an opportunistic microsporidian parasite that can affect a number of different species of mammalian animals and humans. The parasite can pose also threat for rabbits even though it causes several sporadic and asymptomatic infections. Infection of eyes is common and clinical symptom of ocular infection may include uveitis and cataracts. We found out subacute findings in naturally infected animals and show here a first described eye lesions as well as central nervous system and kidneys in Turkey.

**Methods:**

The rabbits (n:171) of breeding units were observed to daily clinical examination for infection of *E. cuniculi* during three years. The eyes of five rabbits (2.9%) showed white intraocular masses or cataracts in the breeding units during daily examinations. The infection was described clinicopathologically in collected organ samples in the animals. During observation, macroscopically, corneal lesions and opacity and impaired lens were taken into attention as well as hyperemia in central nervous system and kidney. Histopathologically, parasitophorous vacuoles pertaining to *E. cuniculi* were detected in all three tissues during different routine Haematoxylin-Eosin and Gram stainings.

**Results:**

Degenerative and necrotic changes in epithelium of cornea and lens and also neurons and tubules were predominantly observed in addition to nonpurulent interstitiel nephritis and encephalitis.

**Conclusion:**

The results from study lead to subacute findings especially in eye during natural *E. cuniculi* infections following asymptomatic and latent changes among breeding colony. The lesions indicated sub-acute stage of *E. cuniculi* infection in eye lesions of rabbit in Turkey.

## Introduction

The obligate intracellular Microsporidium *Encephalitozoon cuniculi* is a zoonotic opportunistic parasite that cause serious disease encephalitozoonosis in animals and people (1, 2) but it is primarily seen in rabbits. Adult rabbits are infected by ingestion of food or water contaminated by *E. cuniculi* spores that are sporadically passed in the urine of infected animals. However, a trans-placental and respiratory route of infection has been reported in rabbits, too (1–4). Rabbits suffering from encephalitozoonosis progress latently asymptomatic and chronically (5). Sudden deaths can follow either asymptomatic process or acute or chronic clinical signs. The main lesions develop neurological signs, renal failure and eye lesions (3, 4).

Macroscopically, there is coincidentally lesions in all organs. It can sometimes include focal, irregular, foci appearance and dents from the surface in kidney in relationship with nonpurulent interstitial nephritis (5, 6). In central nervous system (CNS), findings associated with diffuse nonpurulent meningoencephalomyelitis are observed (5). Histopathologically, early renal lesions are often related with focal to segmental granulomatous interstitial nephritis. Sometimes, ovoid spores may be evident within cells or free form in tubules in medulla renalis. Ongoing stages are only related to interstitial fibrosis (7). In the eye, the anterior lens capsule of eye can rupture spontaneously due to inflammation. Phacoclastic uveitis occurs because of lens capsule rupture together with perilenticular fibroplasia. It causes a zonal granulomatous lens-induced uveitis. The parasite may be seen within the liquefied lens cortex (8–11).

In Turkey, animal encephalitozoonosis has first been described histopathologically (12) and later in rabbits using the carbon immuno assay (CIA) test (13,14). On the other hand, there have been no reported ocular lesions in naturally infected *E. cuniculi* rabbits so far in Turkey. Rabbits were infected with *E. cuniculi* in a breeding colony (14). Rabbits of the breeding colony were clinically examined to prevent the disease during three years according to our study reported serological *E. cuniculi* infection.

We aimed to reveal rarely and naturally occurred sub-acute infection with *E. cuniculi* of rabbits in a breeding colony during observation. Clinically, infection in an eye of rabbits showed white intraocular masses or cataracts in the breeding units during daily examinations. It was encountered with some different histopathological results in mainly central nervous system, eye, and kidney. Sub-acute phase reactions in all those organs might be occurred in some natural encephalitozoonosis.

## Materials and Methods

### Animals and Clinical Examination

The rabbits were breed in a breeding facility in 2012–2015. New Zealand White (NZW) Strain rabbits (n:171) were kept in individual cages with the room temperature set to 20°C and with 12 h light/12 h dark schedule in the breeding facility. The rabbits were fed with a special rabbit pellet diet ad libidum. Special attention was paid to each rabbit; daily clinical examination such as general behaviour, body condition, hydration status, food and water intake for weight loss, reduced appetite leading to anorexia were examined. The animals were observed to the most common clinical parameters such as neurological signs (paresis or paralysis of one or both hind legs, head tilt, seizures behavioural changes) and ocular lesions according to what is reported by previously studies of encephalitozoonosis in rabbits (1, 4, 6, 8–11, 15). Eye lesions of rabbits observed white intraocular masses or cataracts in the breeding units during rutinely veterinary clinical practices. The autopsy was carried out to histopathological examination and definite diagnosis of the eye lesion.

The animal care and pratices were carried out accordance with National Animal Experimentation Ethics (No Article -2b)

### Pathological Process

The autopsy was applied to histopathological examination and definite diagnosis of the eye lesion. After providing sedation and succinyl colin injection, cervical dislocation was performed for each affected animal. Eye lesion of rabbits was marked as EC-1-2-3-4-5. Necropsy was performed systematically and all organs and tissues were examined routinely. Later, the lesions were photographed. The tissue samples were taken from mainly affected central nervous system, eye, and kidney for histopathological examination.

### Macroscopical examinations

Routine methods were used to open the skull. Calvarium and total brain were removed. Cerebrum, cerebellum, medulla oblongata and pons were examined. Both eyes were getting rid of peripheral connection in orbital fossa. Kidneys were removed by cutting peripheral ligaments after removing digestive system and liver. All organs together with those were evaluated according to the general macroscopical evaluation criteria.

### Histopathological examinations

The tissues were fixed in 10% buffered formalin solution, processed in automatic tissue processor and embedded in paraffin. Paraffin blocks were cut with sections of 4 μm in thickness except for central nervous system (6 μm in thickness). Then, they have stained with haematoxylin-eosin (H&E) staining method for the examination of pathological changes. Ziehl-Nielsen and Gram staining methods were used for revealing parasites and PAS staining was used for determination of anti-basal membranous glomerulonephritis and fibrin cap involving larger vessels in other organs.

## Results

### Clinical Examination

**Fig. 1:**
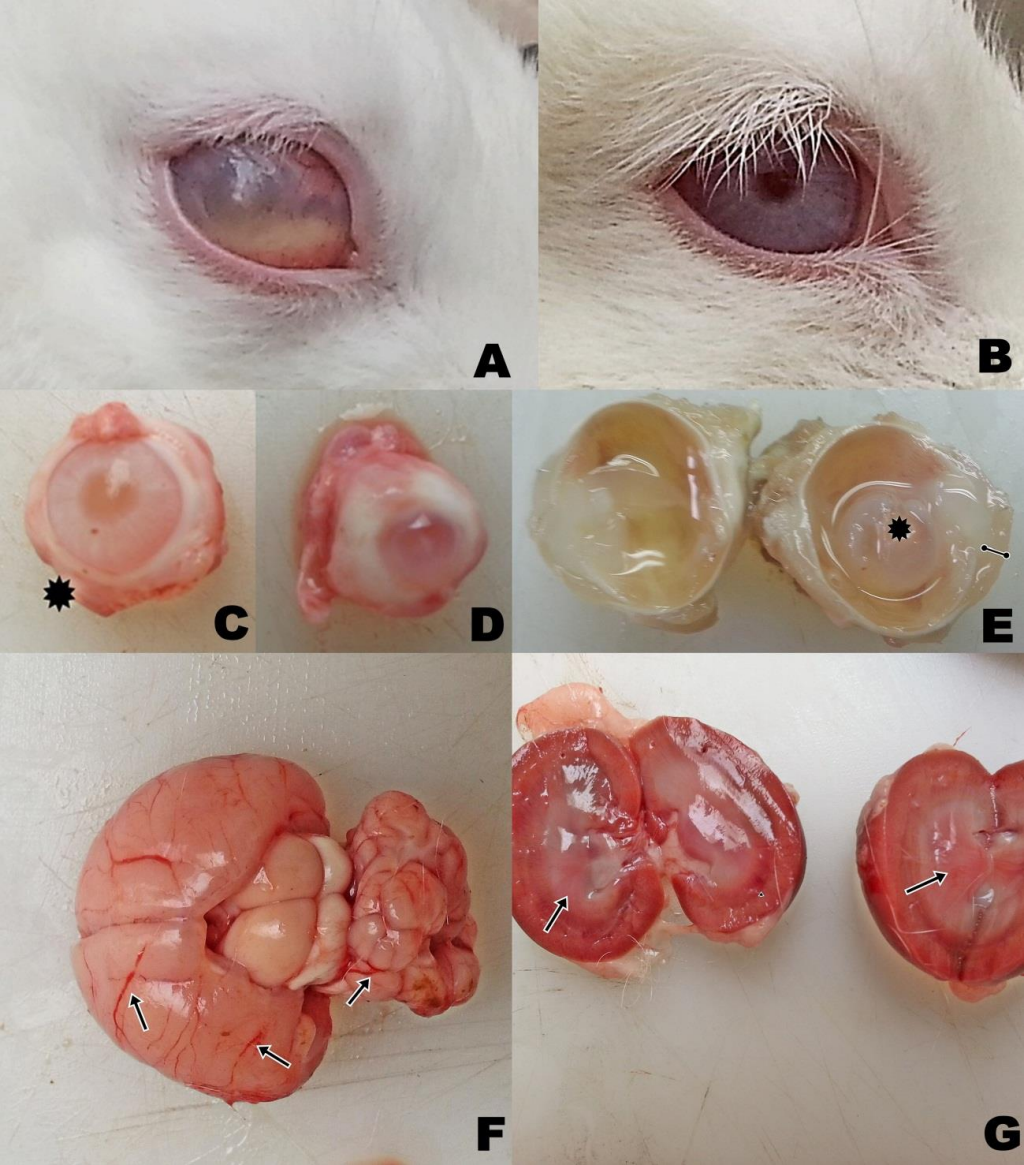
Unilateral ocular lesion of encephalitozoonosis detected in the animals. Affected eye enlarged with cloudy cornea and detected intraocular white masses (A). Normal eye (B). Macrophtalmia in right eye (asteriks) (C-D). Lens opasification (asteriks) and corneal thickness (bar) (E). Congested vessels in cerebrum and cerebellum (arrows) (F). Hyperemia in medulla (arrow head) and pelvis renalis (arrow) of kidney’s cut section (G) (Original)

In rabbits, daily examination parameters such as activities, behaviour, body condition, appetite were normal. Our daily observations and examination of rabbits related to encephalitozoonosis showed no any abnormal clinical findings apart from ocular lesions (Fig. 1). The eye lesions of rabbits were found in five rabbits (2.9%) in the breeding colony. The affected eyes of rabbits showed white intraocular masses or cataracts, and this condition was always found in one eye only. Confirmation of the diagnosis of ocular lesions was performed by histological examination of the eye and also CNS and kidney.

### Macroscopical Findings

Meningeal and cerebral vessels were hyperemic. Mainly cerebrum and cerebellum were edematous. Both kidneys, especially medulla and pelvis renalis, were hyperemic. In eyes, edema was prominent in especially one of animals in cornea. In another animal, microphthalmia was seen in its right eye. In all animals, lenses were subluxated and in three of them, the lenses were emulsified (Fig. 1).

### Histopathological Findings

In central nervous system, there were parasites intracellularly in endothelial cells of meningeal vessels in two animal (EC-1, 4). Cytoplasm and nuclei of pyramidale externum and internum cells degenerated or both were necrotic in neocortex in four animals (EC-1, 3, 4, 5). Moreover, lymphocytic infiltration and gliosis occurred in OCortex and corpus callosum in the other two animals. In hippocampal region, Virchow-Robin spaces of CA1 to CA3 was enlarged in one animal in these were parasite in its vessels (EC-1, 5).

**Fig. 2:**
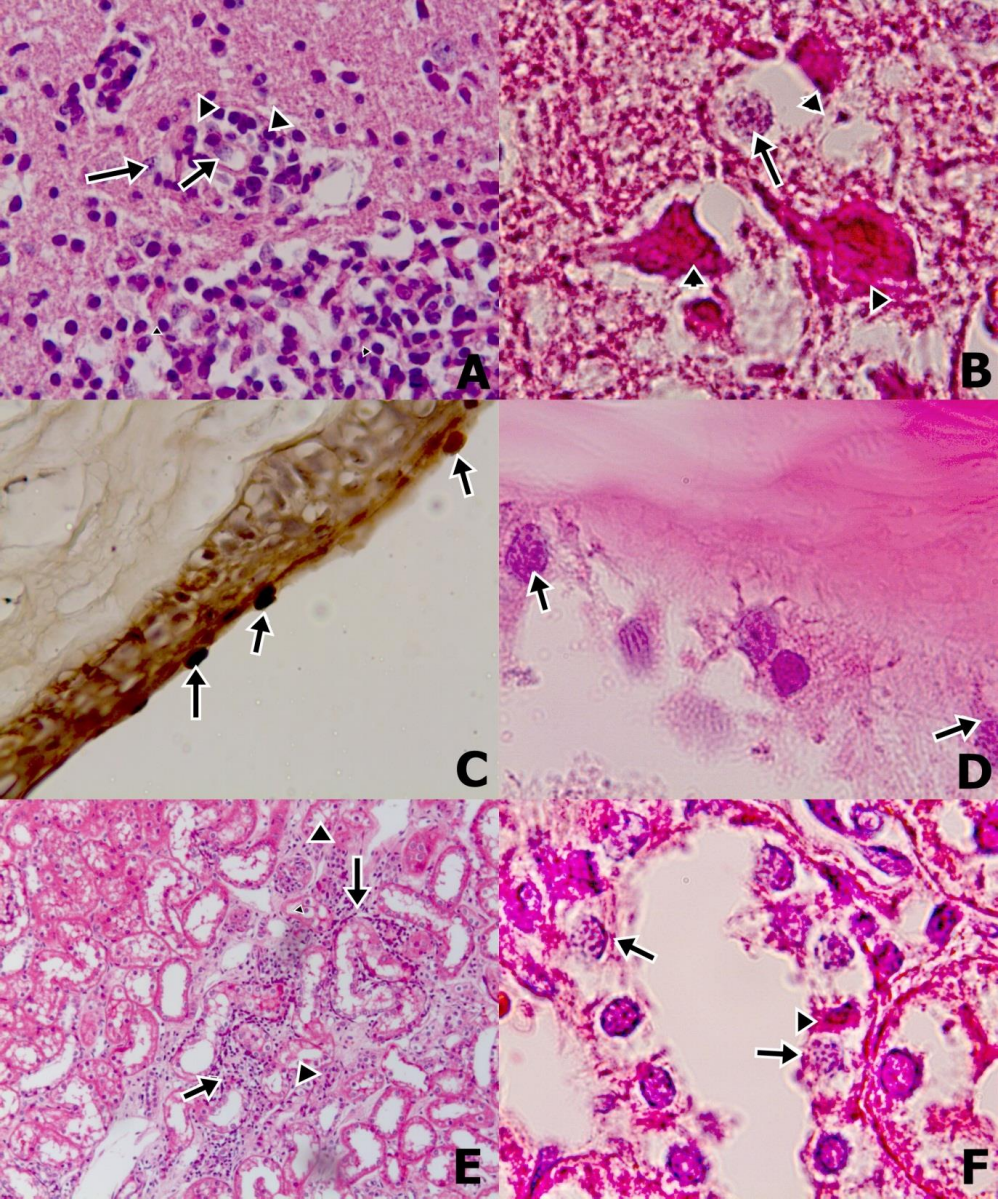
All changes in tissues were evaluated under light microscopy (Euromex). All slide images were obtained by digital photography. Focal gliosis and lymphocytic infiltration (arrowheads) and parasite (arrows) in cerebral cortex, cerebrum, ×400, H&E (A). E. cuniculi spores in parasitophorous vacuole (arrows) and degenerated neurons (arrowhead), substantia grisea in brain (B). Parasites attached at corneal epitheliums, eye, ×400, Gram staining (C). Degenerated Lens Epithelial Cells (LECs) and impairment in lens crystallina (asteriks), ×100, H&E (D). Mononuclear cell infiltration at interstitium (arrows) and degeneration in tubul epitheliums (arrows), ×100, H&E (E). E. cuniculi spores in parasitophorous vacuole (arrows) and picnotic nuclei of tubul epithelium (arrowhead), cortex of kidney ×1000, H&E (F)(Original)

Granular and gangliosum cells were necrotic in especially CA3 and Dentate Gyrus (DG) regions in four animals (EC-1, 3, 4, 5). CA2 cells were impaired in one of them. Ependymal cells lining to lateral ventricles degenerated in two of animals (EC-2, 3). There were parasites located periventricular together with gliosis in one animal (EC-1). In cerebellum, neuropils were decreased in three animals (EC-3, 4, 5). Stratum moleculare, granulosum cells, and Purkinje cells lost their chromatin of nuclei in all animals. Some of Purkinje cells were also necrotic in four of animals (EC-2, 5). Free parasites were observed between especially stratum granulare cells comprising others in one animal (EC-3). There were multifocal demyelination areas having wavy edges in substantia alba in all animals. Neurons in hypothalamus, caudex cerebri, pons and medulla oblangata were generally necrotic in all foci of all animals (Fig. 2 A–B) (Table 1).

**Table 1:** Scoring of lesions in central nervous system in terms of degenerated-necrotic cells in 10 High Power Field (HPF). 0 HPF: No lesions, 1–3 HPF : + (mild), 3–7 HPF: ++ (moderate), 7–10 HPF: +++ (strong)

***Central Nervous System***	***Animal Code and Scoring of lesions***				
	**1**	**2**	**3**	**4**	**5**
	**Cerebrum**				
Neocortex	+++	+++	+++	+++	++
Corpus callosum	++	++	+	+	−
Hypocampus	+++	+++	+	++	−
Thalamus	++	+++	+	+	+
Caudex cerebri	+	+	−	−	−
Pons	+	−	−	−	−
Medulla oblangata	+	−	−	−	−
Cerebellum	+++	+++	+++	+++	+++

There were eye lesions in all animals excepting one animal (EC-5). They were located in cornea, corpus ciliare, and lens. Superficial, suprabasal cells in three animals (EC-1, 3, 4) and included basal cells in an animal were generally degenerated (EC-2). However, some of cells were necrotic. In one animal, cornea epithelium was honeycomb in appearance and there were some microcystic areas in two animal (EC-1, 2). In four animals, large and focal lymphocytes and plasma cell infiltrations and also eosinophilic leucocytes in an animal were encountered in corneal stroma (EC-2). Some of epithelial cells lining corpus ciliare were degenerated and necrotic together with parasites in an animal (EC-2). Capsule and lamelle of lens were impaired, lens stroma and lens epithelial cells (LECs) degenerated in three of animals (EC-2, 3, 4) (Fig. 2C–D).

In kidney, findings were generally focused on entity of parasite and degenerative-necrotic changes in tubular epitheliums. Cytoplasms of mainly cortical and medullar tubules degenerated. In lumina of cortical tubules, there were proteinous ultrafiltrat and hyalin cylindres. On the other hand, many glomerules were hyperemic, hypercellularized and fenestrated in appearance. Bowman spaces of three of animals were enlarged (EC-1, 3, 4). In two animals, focal lymphoplasmacytic infiltrations were also attended in interstitial tissue (Fig. 2E).

All parasites were stained as gram-positive with Gram’s staining method free within a focus of inflammation in corneal stroma in eye, neuropil and granulare layer cells of brain. Or, they are located in within intracellular parasitophorous vacuoles in tubular epithelium and glomerular endothelial cells, corpus ciliare epithelium, ependym cells, and meningeal endothelium (Fig. 2F).

## Discussion

Predilection sites mentioned for the disease, like brain, kidney, and eye, might be histopathologically affected from the parasite (1, 5, 6, 8, 16). In general, the damages in encephalitozoonosis mostly occurs in brain and kidney. Apart from these, eye, liver and also heart can be affected. Parasite selectively located in vascular endotelium causes segmental vasculitis. Vascular involvement leads to hepatic degeneration and focal hepatitis in liver and myocardial necrosis and inflammation in heart (17). In kidney, medulla, and pelvis renalis are the main foci. In early stages of the disease, nonpurulent interstitial nephritis has been described. The numerous parasites are located in glomerular capillaries, tubular epithelium and lumina, interstitium and renal vessels. Fibrinoid necrosis and arteritis can be developed at the end of process (17). During later stages, glomerular fenestration appearance might be observed due to accumulation of antigen-antibody complexes against the parasites. In the presented study, the kidney lesions predominantly involved in that fenestration are degenerative and necrotic changes. Glomerular fenestration and nonpurulent interstitial nephritis reminded that the infection in all animals was in its late stage. Degenerative and necrotic changes in tubules were begun as the result of vascular damages even though there is no severe vasculitis.

About eye, unilateral phacoclastic uveitis together with other eye lesions such as hypopyon, cataracts were observed in a high percentage in almost all infections (1, 9). Regarding infection of *E. cuniculi*, there is no report for phacoclastic uveitis in eye so far (8, 10, 11). The encountered eye lesions in our study were just restricted in cornea, corpus ciliare, and lens. This study might be commented on a different finding. Therefore, the uveal lesions may rarely occur at the terminal stages of the infection. On the other hand, reticular degeneration, microcysts, and microphthalmia, cell accumulation in corpus ciliare are considered as the new findings in such kind of infections. Additionally, microphthalmia might have occurred because impairments of corpus cilare known as humor drainage point of eye, it was restricted with cellular infiltration and increasing in intraocular pressure.

Central nervous system findings are composed of nonpurulent meningoencephalomyelitis, focal gliosis and perivascular mononuclear cellular infiltration in paranchyme and meningeal vessels (17). In our study, the location of lesions was described in detail. Cerebral cortex, hippocampus, and cerebellum were found different from other locations, especially in subacute stage. Meninges, leptomeninges are generally affected from the vasculitis. Besides, second is different finding in our study, there is no meningeal vessel inflammation even if parasites are located in meningeal endothelium in two animals. On the other hand, third one is granulomatous inflammation including central necrotic area and peripheral mononuclear cell infiltrations composed of lymphocyte, plasma cells and glial cells (1, 18, 19); however, it was not encountered with this granuloma in any area of brain. The only thing is focal necrotic areas and degenerative cells were present. There can be very sparse or even absent organisms in these chronic infections (20). However, in our study, parasitophorous vacuoles were seen in mainly substantial grisea. The third different thing was demyelination. There has been no identification regarding demyelination of cerebellum until now. However, demyelination areas were observed with wavy edge in mainly substantia alba of cerebrum and cerebellum in our study. Fourth one was severe cellular infiltrations. In contrast to earlier studies, inflammatory cell infiltration was not taken into consideration any part of central nervous system. However, multifocal gliosis were widespread. The cells might show beginning of repairment in myelin sheets as result of cleaning the debris out in impaired areas. Therefore, those findings can be begun as subacute stage of infection.

Lesion foci in even most specific organs for the infection cannot be occured properly. In this condition, waiting for turning the specific lesions appear might be useful. In our study, naturally *E. cuniculi* infection from the encountered lesions in different organs might so proceed in contrast to being presented seropositivities in all animals. Another difference in our study from before, eye lesions, especially lens lesions, were rather prominent in relationship with proceeding of infection. Findings in developing of subacute stage of the infection might be different from experimental studies.

## Conclusion

The obtained findings in all three organs from our study were partly compatible with previous reports. However, the acute findings already occurred in all the animals. In such natural infections, the lesions for ongoing stages are difficult to forecast. In our study, the lesions indicated sub-acute stage of *Encephalitozoon cuniculi* infection in eye lesions of rabbit in Turkey never described so far. The findings are going to helpful for researchers focusing on naturally occurred *E.cuniculi* infection.
